# How quantitative is metabarcoding: A meta‐analytical approach

**DOI:** 10.1111/mec.14920

**Published:** 2018-12-07

**Authors:** Philip D. Lamb, Ewan Hunter, John K. Pinnegar, Simon Creer, Richard G. Davies, Martin I. Taylor

**Affiliations:** ^1^ School of Biological Sciences University of East Anglia Norwich UK; ^2^ School of Environmental Sciences University of East Anglia Norwich UK; ^3^ Cefas Lowestoft UK; ^4^ School of Biological Sciences Bangor University Bangor UK

**Keywords:** biomass, high‐throughput sequencing, meta‐analysis, metabarcoding, next‐generation sequencing

## Abstract

Metabarcoding has been used in a range of ecological applications such as taxonomic assignment, dietary analysis and the analysis of environmental DNA. However, after a decade of use in these applications there is little consensus on the extent to which proportions of reads generated corresponds to the original proportions of species in a community. To quantify our current understanding, we conducted a structured review and meta‐analysis. The analysis suggests that a weak quantitative relationship may exist between the biomass and sequences produced (slope = 0.52 ± 0.34, *p* < 0.01), albeit with a large degree of uncertainty. None of the tested moderators, sequencing platform type, the number of species used in a trial or the source of DNA, were able to explain the variance. Our current understanding of the factors affecting the quantitative performance of metabarcoding is still limited: additional research is required before metabarcoding can be confidently utilized for quantitative applications. Until then, we advocate the inclusion of mock communities when metabarcoding as this facilitates direct assessment of the quantitative ability of any given study.

## 1 INTRODUCTION

Metabarcoding, the use of a polymerase chain reaction (PCR) and high‐throughput sequencing (HTS) to characterize organisms present in a sample, has been used to address an array of ecological questions (Creer et al., 2016) (PCR‐free sequencing is an emerging technology (Paula et al., 2015; Srivathsan, Ang, Vogler, & Meier, 2016) but is not the focus of this analysis). For example, metabarcoding has allowed the taxonomic identification of many specimens simultaneously using a standardized DNA region (Valentini, Pompanon, & Taberlet, 2009) without the need for on‐the‐ground taxonomic expertise. Similarly, environmental DNA (eDNA) studies, which sequence DNA in soil and water (Yu et al., 2012) without first isolating any organisms, facilitate rapid biodiversity monitoring with only small sediment or water samples. Metabarcoding has also played an important role in uncovering diets and resolving food webs (Pompanon et al., 2012), as well as reconstructing community dynamics temporally using ancient DNA preserved in sedimentary layers (Thomsen & Willerslev, 2015).

**Figure 1:**
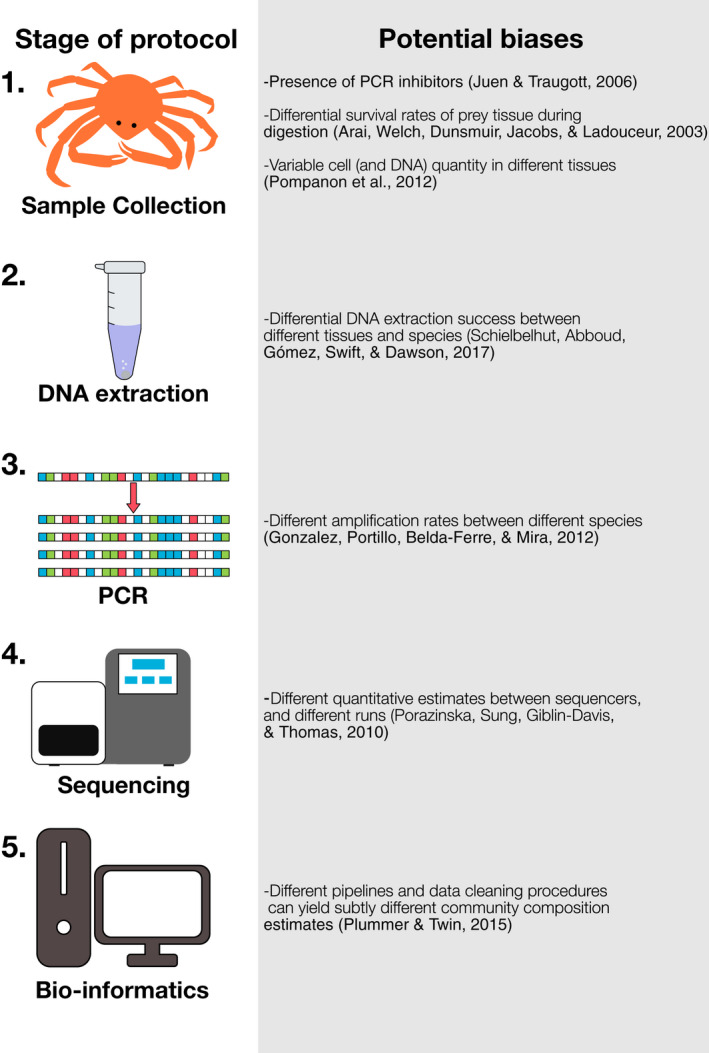
Overview of HTS procedure and factors that can influence the quantitative output [Colour figure can be viewed at wileyonlinelibrary.com]

Early adopters of metabarcoding were hopeful that outputs would be quantitative; that is, that reads obtained from a sequencing run would correlate with biomass in the original sample (Symondson & Harwood, 2014) in a similar manner to other applications such as RNA sequence analysis (Mohorianu et al., 2017) and the characterization of microbial communities (where it is referred to as meta‐genomics). However, several factors, detailed in Figure 1, can introduce bias into the results and yield inaccurate biomass estimates. Yet, despite these factors being well documented, after more than a decade of use there is no clear consensus as to what extent metabarcoding is quantitative. Many studies report their findings in a quantitative manner where the relative read abundance (RRA) (Deagle et al., 2018) is interpreted as the relative abundance of biomass (Kowalczyk et al., 2011; Soininen et al., 2015; Sousa et al., 2016; Vaz et al., 2017). Others use a frequency of occurrence (FOO) approach, also referred to as weighted occurrence (Deagle et al., 2018), where the proportion of samples in which a given sequence was detected is used to infer a different sort of quantitative measure (Bohmann et al., 2011; De Barba et al., 2014). It is also common to incorporate a qualitative approach (detected/not detected), sometimes simply referred to as occurrence (Deagle et al., 2018) or a “species list,” alongside these quantitative approaches (Vesterinen et al., 2016).

Empirically determining the extent to which metabarcoding is quantitative should be relatively simple: take a mixture of organisms with known biomass, PCR and sequence, then compare the results of the HTS run to the original biomass of each community member. Indeed, many studies have used this approach (Leray & Knowlton, 2017; Piñol, Mir, Gomez‐Polo, & Agustí, 2015). However, often only one primer set is used and the output may be a result of primer bias (the differential amplification of target DNA due to different numbers of nucleotide mismatches between the primer and target DNA between samples) rather than a reflection of the ability of metabarcoding techniques. Even if multiple primers are used, they are normally used on the same sequencing run, in which case results cannot be considered independent. An experiment featuring enough sequencing runs to gather sufficient statistical power to disentangle the various factors that may affect quantitative performance would be prohibitively expensive for most research groups. Consequently, there is an ad hoc collection of methodologies that provide different levels of quantitative performance, but little certainty as to whether the variance is due to unique parameters in the experimental set‐up or a result of more general drivers.

In this study, we aim to address this knowledge gap. A structured review was conducted to collate our knowledge about the extent to which metabarcoding for taxonomic assignment is quantitative. Subsequently, a meta‐analysis was conducted to investigate the degree to which metabarcoding is quantitative across multiple independent studies. Factors affecting the quantitative performance such as platform choice, the experimental set‐up (does using biomass, individuals or DNA as the input unit affect quantitative estimates?) and the number of species incorporated in a study were also investigated. Factors that could not be addressed are also discussed to direct future research.

## 2 METHODS

### 2.1 Search strategy

Articles that used quantified multispecies assemblages, PCR and HTS platforms for taxonomic assignment with metabarcoding were targeted using specific search terms. Identifying optimized search terms was important since metabarcoding is now widely used across evolutionary, ecological and medical research. After assessing a variety of search terms, an appropriate combination was finalized: the Web of Science was searched on 31/10/2017 for English language articles for all available years using the following search terms: ((quant* OR diet OR biomass) AND (barcod* OR metabarcod*)). In total, 1,262 articles were retrieved.

### 2.2 Article screening

Initial filtering of the articles was based on their titles: any articles that obviously had no relevance to quantification of biomass using metabarcoding were discarded. After initial filtering, 262 articles remained. These articles were manually inspected, and any that included a quantified community of biomass, individuals or DNA as starting material and reported the proportion of reads obtained from a HTS platform were used for data extraction. Since the slope of a fitted linear model was to be used as an effect size (see below), variation in the amount of input material was also required (equal amount of starting biomass could not be used). In total, 22 articles (Table 1) were used in the meta‐analysis.

**Table 1:** Articles included in the meta‐analysis

Author	Species per trial	Sequencer	Starting material	Organisms	Marker
Albaina, Aguirre, Abad, Santos, and Estonba (2016)	6	454	Biomass	Marine invertebrates (crustaceans, annelids)	18s
Blanckenhorn, Rohner, Bernasconi, Haugstetter, and Buser (2016)	4 to 9	Illumina	Biomass	Macroinvertebrates (coleoptera, diptera, hymenoptera)	COI
Bokulich and Mills (2013)	12	Illumina	DNA/RNA	Yeast	ITS
Deagle, Chiaradia, McInnes, and Jarman (2010)	3	454	Biomass & Faecal	Fish	16s
Diaz‐Real, Serrano, Piriz, and Jovani (2015)	3	454	Individuals	Feather mites	COI
Egge et al. (2013)	11	454	DNA/RNA	Haptophytes	18s
Elbrecht and Leese (2015)	52	Illumina	Biomass	Macroinvertebrates (freshwater)	COI
Elbrecht et al. (2016)	52	Illumina	Individuals	Macroinvertebrates (freshwater)	COI
Elbrecht et al. (2017)	52	Illumina	Biomass	Macroinvertebrates (freshwater)	16s
Geisen, Laros, Vizcaíno, Bonkowski, and De Groot (2015)	8	454	Individuals	Protist culture	18s
Hatzenbuhler, Kelly, Martinson, Okum, and Pilgrim (2017)	5	454	Biomass	Fish	COI
Hirai et al. (2015)	33	454	Biomass	Copepods	LSU
Iwanowicz et al. (2016)	12	Illumina	DNA/RNA	Plants	ITS
Klymus, Marshall, and Stepien (2017)	11	Illumina	DNA/RNA	Bivalves, gastropods	16s
Kraaijeveld et al. (2015)	6 to 11	Ion Torrent	Individuals	Plants (pollen)	TrnL
Pochon, Bott, Smith, and Wood (2013)	9	454	DNA/RNA	Marine invertebrates (echinoderms, crustaceans, ascidians, molluscs, annelids)	18s
Porazinska et al. (2010)	38	454	Individuals	Nematodes	18s
Rocchi, Valot, Reboux, and Millon (2017)	9	Illumina	DNA/RNA	Fungus	ITS2
Saitoh et al. (2016)	9	454	Biomass	Macroinvertebrates (springtails)	16s, COI
Smith, Kohli, Murray, and Rhodes (2017)	10	Illumina	Individuals	Dinoflagellates	Cyt b, LSU, 18s
Thielecke et al. (2017)	5	Illumina	DNA/RNA	Plasmid constructs	n/a
Thomas, Deagle, Eveson, Harsch, and Trites (2016)	3	Illumina	Biomass	Fish	16s

### 2.3 Data extraction

The composition of the community assessed (either biomass, number of individuals or concentration of DNA) and the proportions of reads corresponding to the relevant species in the test community obtained from the sequencing platform were recorded for each trial within an experiment. The sequencing platform, number of species used and the source of input material for each trial within any given study were recorded. The main manuscript and Supporting information were inspected: if possible, data were taken from a table, and if tables were unavailable, the data were manually extracted from figures using *Web Plot Digitizer* (Rohatgi, 2017). If data were not presented in the main article, the corresponding author was emailed to obtain the data.

**Figure 2:**
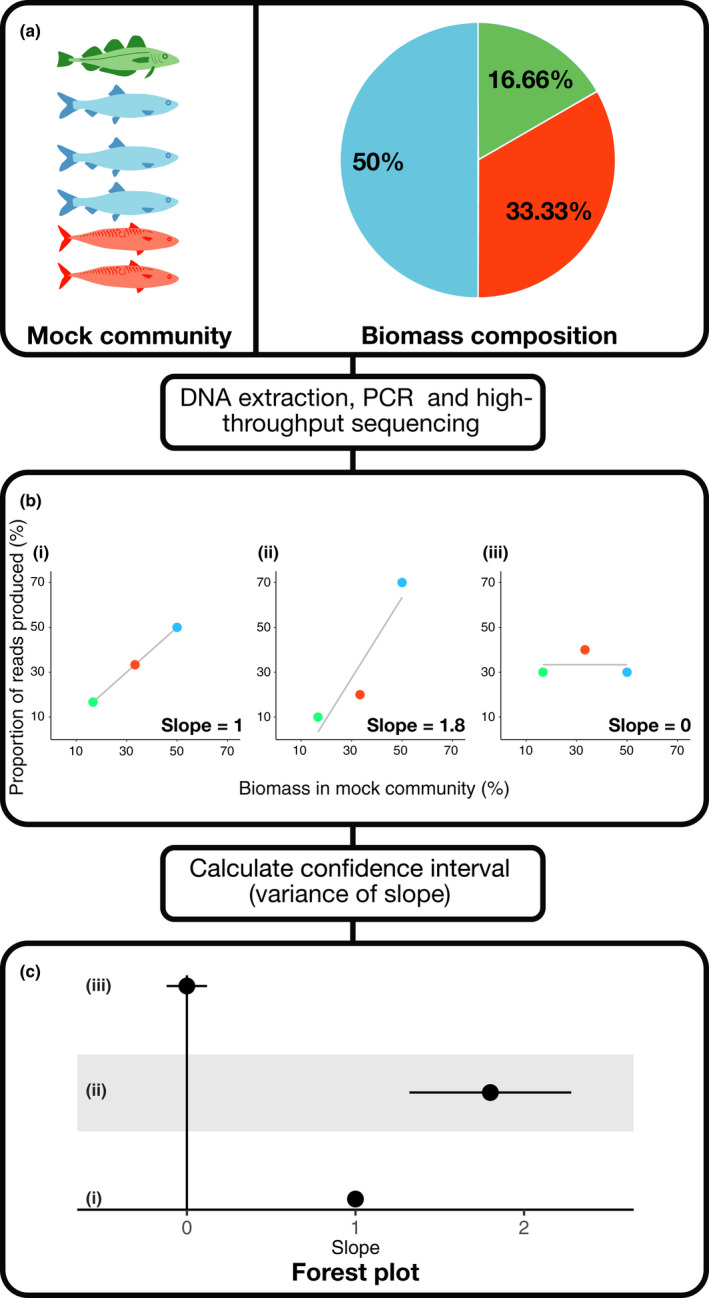
A schematic illustrating how data are utilized in the meta‐analysis. (a) The mock community with quantified biomass. (b) Three hypothetical outcomes of the metabarcoding step: (i) a perfect quantitative relationship between biomass and sequencing yield; that is, a 1% increase in biomass yields a 1% increase in reads, generating a slope = 1. (ii) A quantitative signal in which rank abundance is same in the mock community, but with over‐representation of common sequences and under‐representation of rare sequences resulting in a slope greater than 1. A slope of between 0 and 1 would be produced when common sequences are under‐represented and rare sequences over‐represented (not shown). (iii) No quantitative information, with a slope close to 0. Negative slopes would also be indicative of nonquantitative signals. (c) shows how (i),(ii) and (iii) would be visualized in a forest plot with corresponding variance of slope denoted by error bars [Colour figure can be viewed at wileyonlinelibrary.com]

The composition of the mock community and corresponding sequence data were converted in percentage values (see Figure 2(a)). For the Elbrecht, Peinert, and Leese (2017) study using individuals of varying sizes (Elbrecht et al., 2017), the composition of individuals in the mock community, and the output of reads, was presented grouped by size (large, medium and small individuals) and unsorted. In this instance, we calculated input and output percentages by the sorted size groupings as this was most similar to the approaches used in other included studies.

Slope is a commonly used effect size when the relationship between two continuous variables is being investigated (Rosenberg, Rothstein, & Gurevitch, 2013). In this instance, it was chosen as it is easy to interpret and meets the statistical assumptions of the meta‐analysis model without transformation (in this instance, because slopes did not approach vertical asymptotes and little skewness was present in the data).

### 2.4 Meta‐analysis model fitting

Slope (the effect size) was calculated by fitting a linear model for each trial detected in the review using *R* (R Core Team 2017), such that the proportion of reads produced from the sequencing run would be a function of the proportion of starting material in the experiment. The variance of the slope was calculated in R and used as the sampling variance as described by Rosenberg et al. (2013). Figure 2 illustrates how the results of a mock community experiment are incorporated into this analysis.

All meta‐analysis was conducted in the customizable, open‐source, meta‐analysis package “metafor” (Viechtbauer, 2010) in R. Many studies used multiple trials within a single study; however, these trials cannot be treated as statistically independent from one another. To account for this nonindependence, a cross‐study slope estimate was determined using a two‐level nested random‐effects model using a restricted likelihood function. Trials within an experiment were nested at the study level. The influence of sequencing platform, and DNA source material, was tested by including them as moderating factors in the model. Terms were iteratively omitted from the model, and AIC was used to select the final model.

Weighting of each study in the meta‐analysis model was determined solely by the number of sequencing runs used in each study (e.g., 1 for 1 run, 2 for 2 runs). However, when multiple trials were conducted within a single study, the weight of each trial was calculated by dividing the number of reads produced for the *trial* by the total number of sequences produced by the sequencing run within the study. This allows different sequencing depths within a single study to be accounted for (using a nested model) while maintaining sequencing runs as independent data points. For example in Saitoh et al. (2016), a single sequencing run was used and a meta‐analysis model study weight of one was assigned. Within this study, there were two trials: the 16 s *trial* produced 45% of the reads; therefore, it accounted for 45% of model weight within the nested model (at the study level).

### 2.5 Sensitivity testing

Assessing publication biases (the increased probability of positive results being accepted for publication) in meta‐analytical models is challenging for nested models. Funnel plots are difficult to interpret: studies cluster together due to statistical dependencies rather than genuine biases (Lau, Ioannidis, Terrin, Schmid, & Olkin, 2006). Egger's regression test (Egger, Smith, Schneider, & Minder, 1997), another commonly used metric, is not supported for nested models in the current version of metafor. Consequently, it was not possible to assess whether publication bias may be present in the data set. However, influential trials in the meta‐analysis were visually identified using hat values, which show the importance of any given trial in relation to the model as a whole (Krahn, Binder, & König, 2013), and plotted against the standardized residuals of the meta‐analysis model.

## 3 RESULTS

Across all studies, a significant (*p* < 0.01) relationship existed between the proportion of input material for each species present and the proportions of sequences obtained from metabarcoding. A large amount of observed variation was due to actual differences in the interstudy slope estimate (*I* ^2^ = 88.5%). Across all studies, an effect size estimate (slope) of 0.52 (±0.34 variance of slope) was identified.

**Figure 3:**
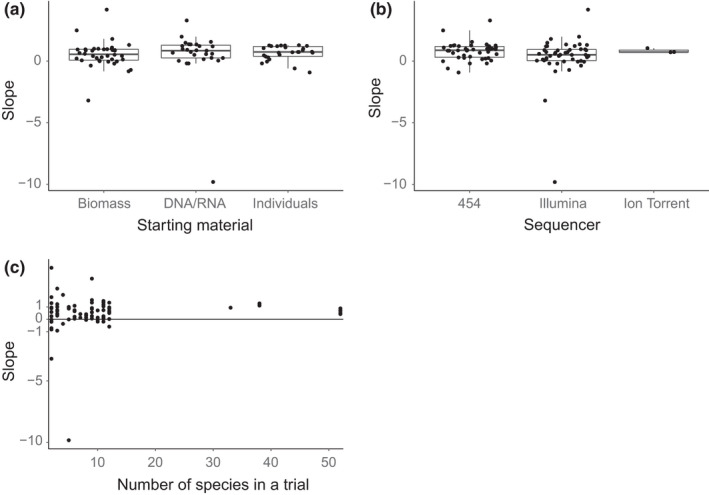
The quantitative ability of metabarcoding using (a) various starting materials, (b) different sequencing platforms and (c) different number of species within in a trial. Note that each point represents a trial, which may not be fully independent from one another. However, this nonindependence is accounted for in the meta‐analysis model

None of the tested moderators, type of sequencer, number of species used in a trial or type of starting material had a significant effect (*p* > 0.05 in all instances) on the estimate provided by the meta‐analysis model. Figure 3 illustrates the lack of difference in quantitative ability (a) between the materials used for metabarcoding, (b) among the sequencers and (c) the number of species used in a trial.

**Figure 4:**
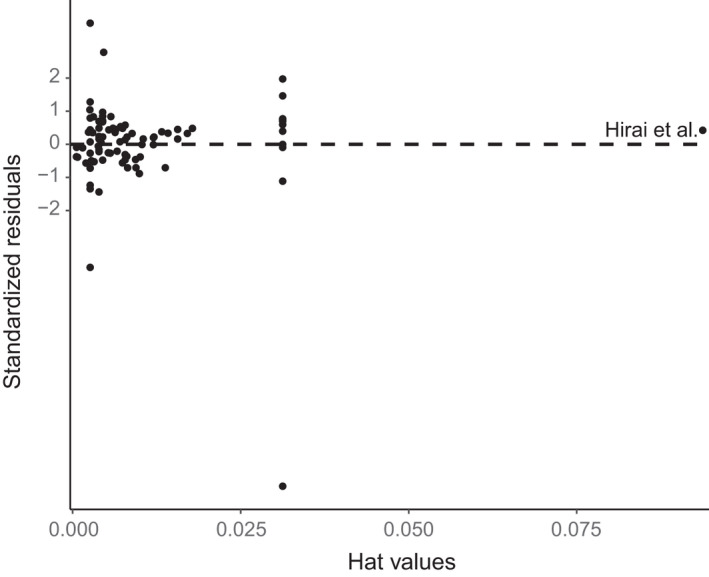
Hat values of trials included within the meta‐analysis (a measure of influence on the meta‐analysis model plotted against standardized residuals). Outlying trials are labelled. Note the points correspond to trial‐level influence, not study‐level influence

Sensitivity testing, using hat values and residuals (Figure 4) appear to show a single trial (Hirai, Kuriyama, Ichikawa, Hidaka, & Tsuda, 2015), was having a large influence on the final output of the model. However, three sequencing runs were used for a single trial in this study, and as such, it has a relatively greater weight in the meta‐analysis compared to most other trials that only used a single sequencing run.

## 4 DISCUSSION

### 4.1 How quantitative is DNA metabarcoding?

**Figure 5:**
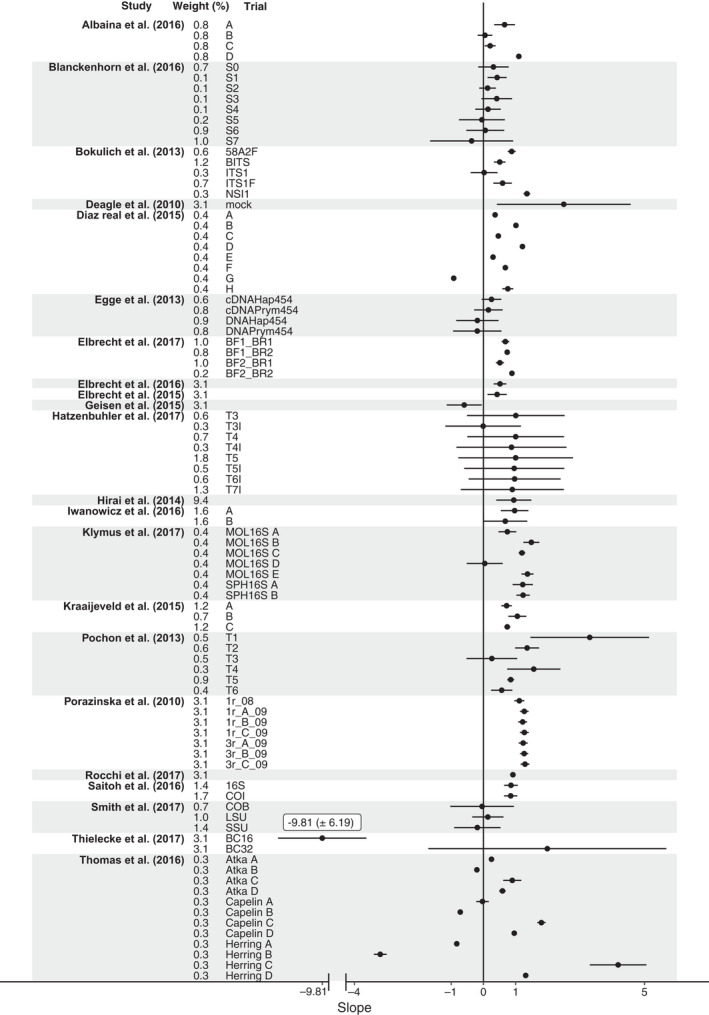
Forest plot showing the slope estimates for all trials in the meta‐analysis (± variance of slope). Trials are clustered at the paper level denoted by the grey and white shading

Across all studies, a slope estimate of 0.52 was identified as the relationship between biomass and sequence read number. This shows that the RRA produced from a metabarcoding loosely corresponds to the relative occurrence of species in the starting material. If no data about composition of a sampled community exist, metabarcoding data interpreted quantitatively could therefore be more informative than treating it in a strictly detected/not‐detected manner even if the accuracy is low. This supports evidence from simulations presented in Deagle et al. (2018), which suggest that a more accurate interpretation of communities can be achieved by treating metabarcoding data quantitatively rather than relying solely on qualitative measures. However, this estimate has a large degree of uncertainty: ±0.34 variance of slope suggests that in real‐world applications, metabarcoding can be either somewhat quantitative or produce a very weak signal. This uncertainty is reflected within some of the experiments themselves: Figure 5 shows that while many of the included trials appeared to produce quantitative results, variance of the slopes was sufficiently large, overlapping with 0, that a nontrivial probability exists that nonquantitative data will be produced on any given sequencing run. Furthermore, there are several trials included in this meta‐analysis in which metabarcoding produced extremely poor quantitative performance. With such variation between studies, and no easy way to diagnose whether any given metabarcoding study has produced quantitative results, it is easy to see how different opinions on the quantitative ability of metabarcoding have arisen. Focusing on the factors influencing the quantitative performance is essential to further clarify this situation.

The influence of sequencing technology and initial experimental design was included as moderating factors in the initial model. The sequencing platforms did not significantly differ in quantitative ability. This was unexpected, as the different platforms have different technical approaches towards sequencing (Mardis, 2008), and different levels of bias were expected. Additionally, Illumina platforms produce many more reads than other platforms, so a greater level of precision might have been expected. This is not to say platform choice is not important when undertaking a metabarcoding study: read length, sequencing accuracy and cost will all play a role in determining the best choice for a given study. However, these results suggest that in terms of attaining quantitative data, any difference between sequencing technologies is too subtle to be detected in this meta‐analysis, and the factors driving quantitative performance perhaps lie elsewhere in the experimental set‐up.

It has been hypothesized that including a higher number of species in a metabarcoding study will improve the quantitative performance as different amplification efficiencies will have diminishing effects on the overall quantitative performance as the number of species used increases (Deagle et al., 2018; Piñol, Senar, & Symondson, 2018). However, this relationship was not detected here. This may be due to most of the included studies using relatively few species: only three studies had more than 30 species. Thus, the lack of relationship may be driven by lack of variation in the data. Additionally, it is expected that different primer sets, or other factors tested here, would explain much of the variation. Our ability to detect subtle trends in a noisy data set is limited with relatively few studies. This relationship could be better characterized with empirical studies, or if the amount of data available for meta‐analysis were to increase substantially.

Different input materials had no explanatory ability in the final model: sequences were able to replicate the original biomass, quantity of DNA or individuals in a study equally well. We believe this may be because counts of individuals were frequently used for species of similar size: if there is little variation in size of individuals between different species, count data can be regarded as a proxy for biomass. A notable exception in using counts of individuals from species of similar sizes was the Elbrecht et al. (2017) study: here, species were sorted by size prior to sequencing. The authors demonstrated that sorting individuals by size affected the quantitative ability of metabarcoding by comparing a mock community sorted by size and a mock community where individual size was not considered. We used the sorted data treatment as this was most similar to other studies in the meta‐analysis. However, given that counts of individuals and biomass were proxies in many studies, and empirical evidence suggests that the RRA does not correspond with the number of individuals if significant size differences are present (Elbrecht et al., 2017), we would advocate caution when inferring count data from metabarcoding data without a priori knowledge of minimal size variation between individuals.

No difference in quantitative performance existed between studies using quantified DNA as a starting material and those that used biomass. Given DNA extraction is the only step (Figure 1) separating these points in the protocol, this suggests it is not a source of significant bias in the studies included in the meta‐analysis. However, it must be noted that this is not always the case: Pornon et al. (2016) reported a 300‐fold difference in DNA concentrations after extraction. It is possible that structural differences in the exine (the tough protective coating of pollen) may have driven the variable DNA yield. Although not a significant factor in this study, best practice would dictate that quantifying the relationship between biomass and DNA yield in the target organisms is advised prior to metabarcoding.

### 4.2 Future directions

This analysis has shed light onto some, but not all, of the factors that influence the quantitative performance of metabarcoding. Although not considered here, primer bias is likely a large source of variation in the quantitative performance of metabarcoding studies: Piñol et al. (2015) empirically tested the relationship between primer mismatch and amplification efficiency and found mismatches accounted for 75% of variation. We had hoped to explore the effect of primer bias on the quantitative performance of metabarcoding by using the nucleotide pairwise diversity at the primer binding site of the mock community as a moderating factor in the final model. Unfortunately, this was not possible: the sequence in the target DNA at the primer site could not be inferred from the studies included in this meta‐analysis as, at most, only the primer sequence can be obtained. For a number of studies, sequences covering the primer binding region were not present in DNA databases. Additionally, even for those species which had relevant sequences, interindividual variation was a concern: amplification efficiency is very sensitive to both the type of nucleotide mismatch between the target DNA and the primer, and the location of the mismatches in the primer sequence (Stadhouders et al., 2010). Without knowing the actual sequence present in the individuals used in the studies, we opted to omit primer site mismatches from this analysis. However, the effect of nucleotide mismatches in primer sequences on quantitative performance of metabarcoding is explored in detail through the use of simulations in this issue (Piñol et al., 2018). This topic will be an ongoing research area, and until we accurately determine the quantitative performance of any given primer set, we would advocate reporting all in silico testing to assess the quantitative ability of primers, and the inclusion of a mock community control on each sequencing run to gauge how accurately RRA corresponds with the starting material.

### 4.3 Reflection on meta‐analysis

It is important to remember what is entailed in a meta‐analysis: a consensus of studies included in the analysis, weighted by sample size. Studies were included based on their detection in a structured review; although this presents a transparent, repeatable, way of including literature, our approach may have missed some relevant studies. Indeed, not all of the high‐quality literature detected in the structured review was included (Leray & Knowlton, 2017; Piñol et al., 2015), due to their experimental design being incompatible with our analytical framework, rather than any shortcomings of the work or relevance to contribute further understanding on the topic.

It should be noted that incorporating results into meta‐analysis necessitates some loss of nuance in the results. Most notably, in this study, we used the slope derived from a linear model as an effect size to facilitate synthesis. However, the quantitative nature of the relationships reported in this analysis may well be more complex than reported by a linear model. As such, we would encourage readers to use this manuscript only as reference material, and assess the cited literature themselves, as a perfect distillation of included literature is inherently not possible.

Furthermore, publication bias remains an unknown factor. Using a nested model to account for nonindependence makes using most common tests for publication bias problematic as they detect the structure implemented in the model, not genuine publication bias. Not accounting for the nonindependence of trials run on the same sequencing run was, we felt, a more immediate flaw than accounting for publication bias. That unfortunately leaves us in a position where the extent of any publication bias is unknown, and we are unable to say how important, or trivial, the issue may be: as such, we reiterate that any synthesis drawn from this model may have been influenced by the omission of unpublished data, as much as the studies included.

Another issue worth considering is the relative weighting given to each study. Meta‐analyses differ from a simple vote count by assigning increased weighting to studies with a larger sample size. Here, weighting was assigned based on the number of sequencing runs used in a study. We feel this weighting is more appropriate than a simple vote count, but it is worth highlighting the results presented here are influenced more heavily by some studies than others; for example, Porazinska, Sung, Giblin‐Davis, and Thomas (2010) had the greatest influence on the model (21.7%) due to the study's use of seven sequencing runs.

Finally, it should be noted this analysis quantifies the understanding of the field at a point in time rather than attempting to be a final point of authority. As highlighted above, much more research is still to be done in this area, and we hope the shortcomings and gaps highlighted will be filled as exciting new research reveals a more mechanistic understanding of this topic.

## 5 CONCLUSION

Our meta‐analysis suggests that metabarcoding possesses some quantitative ability: a cross‐study slope estimate of 0.52 was found, suggesting a weak quantitative signal is present, albeit with a large degree of uncertainty (±0.34 variance of slope). Quantitative ability did not appear to differ among sequencing platforms, the number of species included in a trial or with different starting materials: biomass, individuals or DNA. We remain sceptical that individual count data can be reliably inferred from metabarcoding if there are large size differences between the individuals being assessed and would advise against count‐based inferences without a priori knowledge of the community being assessed. All presented results have probably been influenced by the relatively small sample sizes: additional research is warranted to reveal the mechanistic factors driving quantitative performance. While metabarcoding may eventually become a quantitative tool, many uncertainties remain. Moving forward, we suggest explicitly testing the relationship between read abundance and input biomass using mock communities included as quantitative controls during metabarcoding. Not only will this allow researchers to assess their own study, but it will also assist future meta‐analyses. We also recommend presenting all trials and simulations used in primer selection to make the rationale behind primer choice transparent. Finally, we would encourage additional empirical research into the mechanistic factors behind primer bias in metabarcoding since this is difficult to study using meta‐analytical techniques, yet potentially holds the key to truly quantitative metabarcoding.

## 6 UNCITED REFERENCES

Arai et al. (2003), Gonzalez et al. (2012), Juen and Traugott (2006), Plummer and Twin (2015), Schiebelhut et al. (2017).

## AUTHOR CONTRIBUTIONS

P.D.L conceived the study, conducted review and meta‐analysis, and wrote the first draft of the manuscript. The supervisory team (S.C., E.H., J.K.P., R.G.D. and M.I.T.) critiqued and commented on the analyses and edited the manuscript.

## Data Availability

The data used in this meta‐analysis are uploaded to the Dryad Data Repository: https://doi.org/10.5061/dryad.085jj60.
